# IL-17 mediates inflammatory reactions via p38/c-Fos and JNK/c-Jun activation in an AP-1-dependent manner in human nucleus pulposus cells

**DOI:** 10.1186/s12967-016-0833-9

**Published:** 2016-03-17

**Authors:** Jing-kun Li, Lin Nie, Yun-peng Zhao, Yuan-qiang Zhang, Xiaoqing Wang, Shuai-shuai Wang, Yi Liu, Hua Zhao, Lei Cheng

**Affiliations:** Department of Orthopaedic Surgery, Qilu Hospital, Shandong University, Jinan, 250012 Shandong People’s Republic of China; Department of Orthopaedic Surgery, Shanghai Ninth People’s Hospital, Shanghai, 200011 People’s Republic of China; Qilu Hospital Shandong University, No.107, Wen Hua Xi Road, Jinan, 250012 Shandong People’s Republic of China

**Keywords:** IL-17, COX2, PGE2, LBP, MAPK, AP-1

## Abstract

**Background:**

Low back pain and sciatica caused by intervertebral disc (IVD) disease are associated with inflammatory responses. The cytokine interleukin 17 (IL-17) is elevated in herniated and degenerated IVD tissues and acts as a regulator of disc inflammation. The objective of this study was to investigate the involvement of IL-17A in IVD inflammatory response and to explore the mechanisms underlying this response.

**Methods:**

Cells were isolated from nucleus pulposus (NP) tissues collected from patients undergoing surgeries for IVD degeneration. The concentrations of COX2 and PGE2, as well as of select proteins involved in the mitogen-activated protein kinase (MAPK)/activating protein-1 (AP-1) pathway, were quantified in NP cells after exposure to IL-17 with or without pretreatment with MAPK or AP-1 inhibitors.

**Results:**

Our results showed that IL-17A increased COX2 expression and PGE2 production via the activation of MAPKs, including p38 kinase and Jun N-terminal kinase (JNK). Moreover, IL-17A-induced COX2 and PGE2 production was shown to rely on p38/c-Fos and JNK/c-Jun activation in an AP-1-dependent manner.

**Conclusion:**

In summary, our results indicate that IL-17A enhances COX2 expression and PGE2 production via the p38/c-Fos and JNK/c-Jun signalling pathways in NP cells to mediate IVD inflammation.

## Background

Low back pain (LBP) and sciatica are among the world’s leading causes of disability, constituting 11.9 % of disability cases worldwide [1, 2]. Intervertebral disc (IVD) herniation is considered to be the main cause of LBP and sciatica [3]. Currently, disc herniation has been shown to be involved in a series of inflammatory processes [4–6], and the causes of the IVD inflammatory response have been reconsidered based on a series of cellular and molecular mediators of inflammation [7–11]. However, the precise mechanisms underlying IVD immune-mediated inflammation remain elusive.

Herniated disc tissues express a variety of inflammatory factors, including interleukin-1α (IL-1α) [12], IL-1β [13], IL-6 [14], IL-10[13], nitric oxide[15], tumour necrosis factor-alpha (TNF-α) [16, 17],and prostaglandin E2 (PGE2) [17, 18]. Among these, TNF-α, IL-6, and PGE2 have unique pathological characteristics that are mainly associated with the human body’s immune response. PGE2 is an inflammatory factor that can sensitize nerves and then induce pain [18, 19]. Two enzymes, cyclo-oxygenase 1 and 2 (COX1 and COX2), are involved in the production of PGE2. Unlike COX1, COX2 is an inducible enzyme that might be involved in the pathogenesis of lumbar disc herniation by upregulating PGE2 production. High levels of PGE2 and COX2 have been observed in human herniated disc tissues [10, 18–20], and the resultant inflammatory reactions may cause low back pain or sciatica. Recent studies have revealed that PGE2 production increases in nucleus pulposus (NP) tissues under conditions of low tonicity [21], which may indicate that PGE2 plays an important role in the inflammatory reactions associated with IVD.

Il-17A is a cytokine that is mainly produced by activated Th17 cells and is required for the onset of several diseases, including rheumatoid arthritis [22, 23], asthma[24], inflammatory bowel disease[25] and lumbar disc herniation [5, 26, 27]. Previous studies have revealed that IL-7 can upregulate CCL20 production in NP cells to recruit Th17 cells to herniated disc tissues via the CCL20/CCR6 pathway, which increases IL-17 levels in disc tissue [5]. However, the manner and precise pathway by which IL-17 contributes to immune-mediated disc inflammation remain unclear. Cell metabolism in IVD tissues is mediated by various pathways. Mitogen-activated protein kinases (MAPKs) in chondrocytes and NP cells have received much attention. There are three major MAPKs, p38 kinase, c-Jun N-terminal kinase (JNK) and extracellular signal-regulated kinase (ERK), which participate in different signalling pathways to control cell growth, differentiation and apoptosis [28, 29]. Activator protein-1 (AP-1) is a downstream transcription factor in the MAPK pathway and can interact with specific DNA sequences called AP-1 sites [30]. It has been shown that the MAPK pathway plays a vital role in IVD degeneration [31–34]. Meanwhile, IL-17 was reported to display pro-inflammatory effects by activating the MAPK pathway in nerve cells and tumour cells [35–38].

How IL-17 contributes to the IVD inflammatory reaction is an important question. Identification of the downstream targets of IL-7 in IVD could advance our understanding of the mechanisms underlying IL-17-mediated IVD inflammation. NP cells are known targets of IL-17. In this study, we demonstrate that IL-17 enhances the expression of COX2 and PGE2 via activation of the MAPK/AP-1 pathway in NP cells, which leads to IVD inflammation and the development of LBP or sciatica. These findings may provide a new therapeutic approach for treating IVD inflammation and relieving LBP and sciatica.

## Methods

### Ethics statement

Specimens were collected from patients who underwent lumbar operations between February 2014 and April 2015 in Qilu Hospital of Shandong University, Jinan, China. The present study was approved by the Medical Ethical Committee of Shandong University Qilu Hospital. Written informed consent documents were obtained from all patients involved in this research.

### Isolation and culture of primary IVD cells

Human lumbar IVD samples that would have otherwise been discarded were obtained from patients undergoing posterior lumbar interbody fusion (PLIF) operations or lumbar discectomy surgeries for degenerative diseases (n = 20 patients, aged 21–45). Primary IVD cells were isolated and cultured as previously reported [5]. All of the samples were anonymized, and only patients’ genders and ages were recorded. Disc tissues were transferred to the laboratory immediately. The discs were washed with cold, aseptic phosphate-buffered saline (PBS) to remove residual blood. IVD tissues were carefully separated and cut into fragments of approximately 1 mm^3^. The tissue samples were separately digested with trypsin and type II collagenase (Sigma-Aldrich, Ltd, China).NP cells were filtered through a 200-mesh sieve. The isolated NP cells were seeded as a monolayer and cultured in DMEM/F12 media (Hyclone, Thermo Co., USA) containing 15 % FCS and 1 % PS under standard incubation conditions (37 °C, 95 % air, 5 % CO2, pH 7.2) for approximately 3 weeks. After the primary cells adhered to the bottom of the culture bottle, the culture media was replaced every 3 days [39, 40]. When the NP cells were almost 80 % confluent, the cells were dissociated with 5 % trypsin and subcultured at a ratio of 1:3. Primary NP cells from passages 2 or 3 were harvested and cultured in 6-well plates in which IL-17A stimulation experiments could be performed. When the cells in the 6-well plates were approximately 70–80 % confluent, they were cultured in serum-free medium for 12 h and treated with sterile PBS with or without recombinant IL-17A (PeproTech, USA). After differential cytokine treatment, the supernatants and NP cells were harvested for further analysis.

### Enzyme-linked immunosorbent assay (ELISA)

Supernatants were collected from IL-17-treated and untreated NP cells. We measured PGE2 levels in supernatants from the conditioned NP cell cultures using a human PGE2 ELISA kit (BlueGene, China) according to the manufacturer’s protocol. Colorimetric reactions were read at 540 nm on an Infinite M200 multifunction plate reader (Tecan scientific, Switzerland). PGE2 levels were normalized to total protein levels.

### Total protein and nuclear protein extraction and western blotting

After the indicated treatment, the cultured NP cells were washed with PBS three times and placed in RIPA lysis buffer (Beyotime Biotechnology Co., Beijing, China) supplemented with 5 % PMSF (a protease inhibitor). The cells were scraped from the plates, and the total protein lysates were transferred to microtubes and centrifuged at 15,000 rpm for 15 min at 4 °C. Nuclear proteins were extracted using a nuclear protein extraction kit (Beyotime Biotechnology Co., Ltd., Beijing, China) according to the manufacturer’s instructions. The concentration of each protein sample was determined using a BCA protein assay kit (Beyotime Biotechnology Co., Beijing, China) according to the manufacturer’s instructions. Equal amounts of protein (10 μg) were subjected to 10 % sodium dodecyl sulphate-polyacrylamide gel electrophoresis (SDS-PAGE) and subsequently transferred to polyvinylidene difluoride (PVDF) membranes. The PVDF membranes were blocked in 5 % nonfat milk dissolved in 125 mM NaCl, 25 mM Tris–HCl and 0.1 % Tween 20 (TBST) for 3 h and then probed with relevant primary antibodies overnight at 4 °C, followed by incubation with an immunoglobulin G-horseradish peroxidase (IgG-HRP) secondary antibody (1:2000; Golden Bridge Biotechnology Co., Ltd., Beijing, China) for 1 h. The membranes were also probed with a rabbit anti-GAPDH antibody (1:4000, Abcam Biotechnology Co., USA) or a rabbit anti-lamin B antibody (1:4000, Abcam Biotechnology Co., USA) as a loading control. The following primary antibodies were used in this study: rabbit anti-p-p38, rabbitanti-p-JNK, rabbit anti-c-Jun (all 1:2000; Millipore Biotechnology Co., Ltd., America), mouse anti-p-ERK, mouse anti-c-Fos (all 1:2000; Millipore Biotechnology Co., Ltd., America), rabbit anti-COX2, rabbit anti-p38, rabbit anti-JNK, and rabbit anti-ERK1/2 (all 1:2000; Abcam Biotechnology Co., Ltd., United Kingdom). Protein bands were visualized using a FluorChemE chemiluminescent imaging system (Cell Biosciences, Santa Clara, CA) and quantified by densitometry analysis using Image J software (National Institutes of Health, USA). The integrated density data of target signals were normalized against reference data. The target/reference ratio represents the relative levels of each target protein.

### Immunofluorescence staining

NP cells were transferred to a 24-well plate at a density of approximately 3 × 10^4^ cells/well. The NP cells were cultured in serum-free medium for 12 h and then treated with either PBS or 100 ng/ml IL-17A for 24 h. The cells were then washed with cold PBS three times, fixed with 4 % paraformaldehyde for 15 min, permeabilized with 0.5 % Triton X-100 for 10 min, blocked with 10 % goat serum for 1 h, and incubated with a primary antibody against p-c-Jun (1:400; Millipore Biotechnology Co., USA) overnight at 4 °C. Then, the cells were washed three times and incubated with an anti-rabbit IgG secondary antibody (FITC) (1:100; Golden Bridge Biotechnology Co., Ltd., Beijing, China) for 1 h and 10 μM 4,6-diamidino-2-phenylindole (DAPI) for 15 min at room temperature to stain nuclei. The NP cells were also treated with either PBS or 100 ng/ml IL-17A for 15 min, the cells were incubated with a primary antibody against p-p38 (1:400; Millipore Biotechnology Co., USA) overnight and an anti-rabbit IgG secondary antibody (TMRITC) (1:100; Golden Bridge Biotechnology Co., Ltd., Beijing, China) to perform an immunofluorescence test. The samples were observed using fluorescence microscopy.

### Statistical analysis

All experiments were repeated at least three times, and all data points represent the mean ± standard deviation of three independent experiments. Statistical analysis was performed using a two-tailed Student’s *t* test. A p value <0.01 (denoted with * or ^#^) was considered to be statistically significant.

## Results

### Effects of IL-17A on COX2 and PGE2 production in primary NP cells

**Fig. 1:**
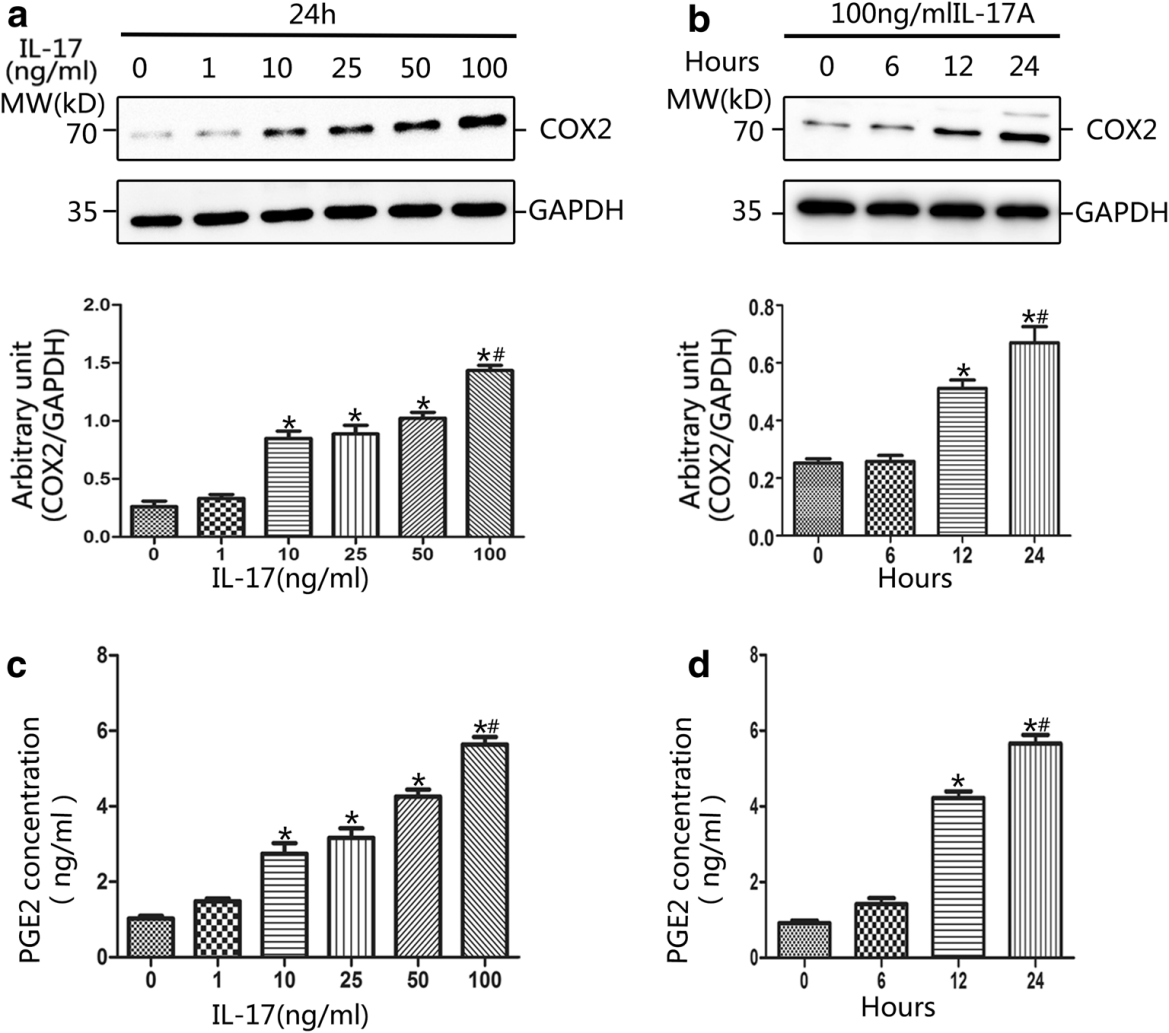
Effect of IL-17A on expression of COX2 and PGE2 in NP cells. **a** IL-17A induced COX2 expression in a concentration-dependent manner. NP cells were stimulated with the indicated concentrations of IL-17A for 24 h. Western blot analysis demonstrated that IL-17A of 10 ng/ml could significantly increase the COX2 expression and the optimal concentration was 100 ng/ml. **b** IL-17A induced COX2 expression in a time-dependent manner. Cell cultures were treated with optimal concentration of IL-17A for 0, 6, 12 and 24 h. Western blot results showed that IL-17A (100 ng/ml) could induce a time-dependent increase of COX2. **c** ELISA analysis revealed that IL-17A induced PGE2 secretion in a dose-dependent manner. The cell-free supernatants were harvested and the expression levels of PGE2 protein were quantified by ELISA. **d** IL-17A induced PGE2 secretion in a time-dependent manner. Regarding to both COX2 and PGE2, there were statistically significant differences between concentrations higher than 10 ng/ml and the control. And, statistical significance was verified of IL-17A treatment longer 12 h. (**a** and **c**: *P < 0.01, vs. control; ^#^P < 0.001, vs. 10 ng/ml; **b** and **d**: *P < 0.01, vs. control; ^#^P < 0.001, vs. 12 h.)

Previous studies have shown that Th17 cells and IL-17A target NP cells in vitro and in vivo [5, 41]. In our study, when exposed to IL-17A treatment, primary NP cells responded by significantly increasing COX2 expression and PGE2 production relative to cytokine-free controls (Fig. 1). IL-17A alone enhanced COX2 expression in primary NP cells from degenerated IVD tissues in a concentration-dependent manner, with an optimal stimulation concentration of 100 ng/ml (Fig. 1a). Furthermore, the optimal concentration of IL-17A was used to treat NP cells for 6, 12 and 24 h, and COX2 expression showed a statistically significant increase at 12 h. An obvious time-dependent effect on the expression of COX2 was also observed (Fig. 1b). Similar concentration-dependent and time-dependent trends were observed for PGE2 secretion from NP cells following stimulation with IL-17A (Fig. 1c, d). In conclusion, IL-17A significantly induced COX2 production and PGE2 secretion in NP cells.

### IL-17A induces activation of the JNK-MAPK and p38-MAPK pathways in primary NP cells

**Fig. 2:**
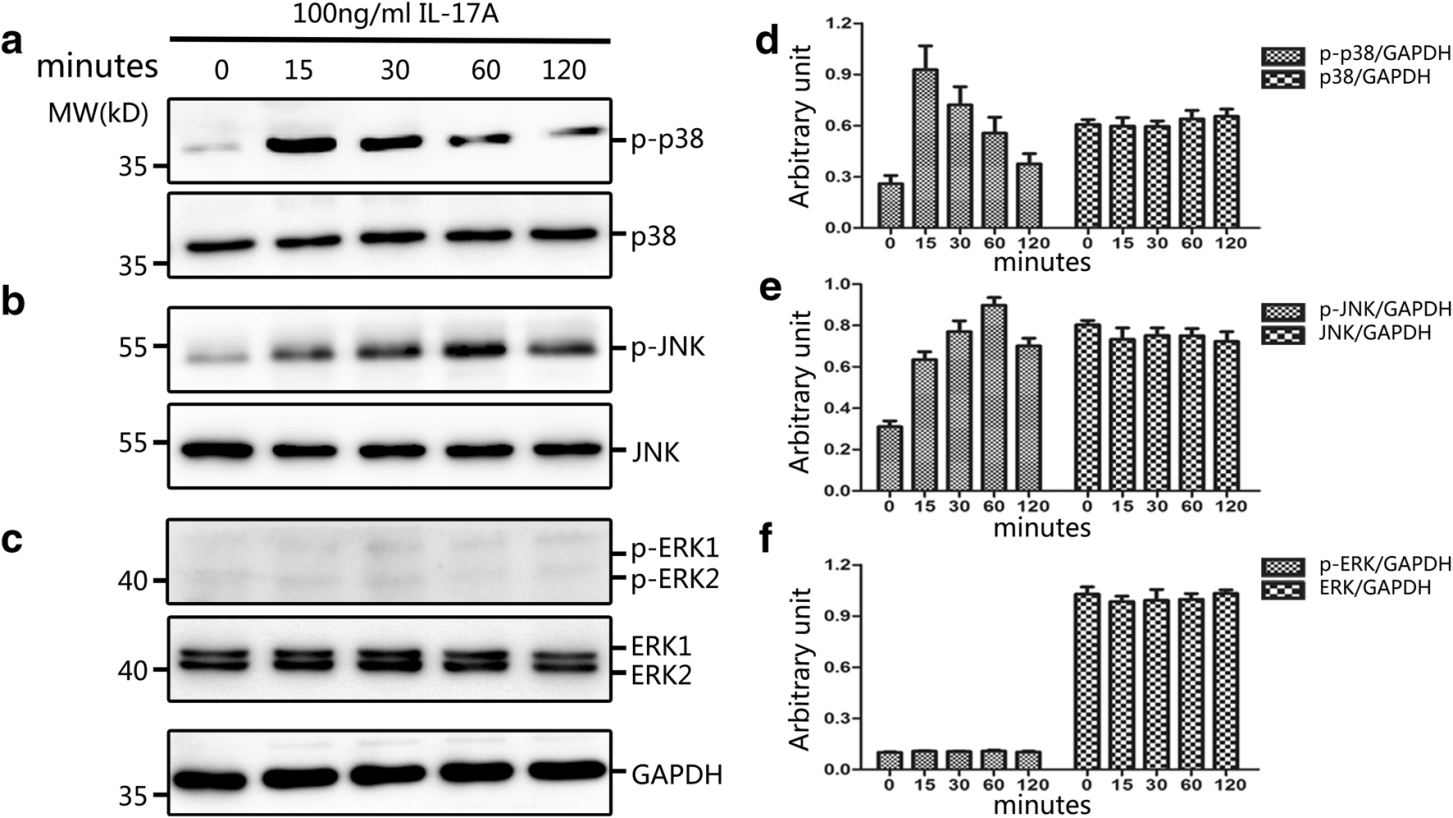
Time course of p38, JNK and ERK MAPK pathway activation by IL-17A. NP cells were cultured in serum-free DMEM-F12 medium for 12 h to synchronize the cells and to make them quiescent before IL-17A stimulation. Immunoblot analysis of total cell extracts isolated from primary NP cells treated with 100 ng/ml IL-17A for various time. Equivalent amount protein (10 μg) were resolved on 10 % SDS-PAGE, electroblotted and immunoblotted using antibodies against (**a**, **d**) the phosphorylated forms of p38 (*upper panel*), or total p38 (*lower panel*); or (**b**, **e**) the phosphorylated forms of JNK (*upper panel*), or total JNK (*lower panel*); or (**c**, **f**) the phosphorylated forms of ERK (*upper panel*), or total ERK (*lower panel*). IL-17A treatment could induce phosphorylation of p38 and JNK, but no obvious phosphorylation of ERK

**Fig. 3:**
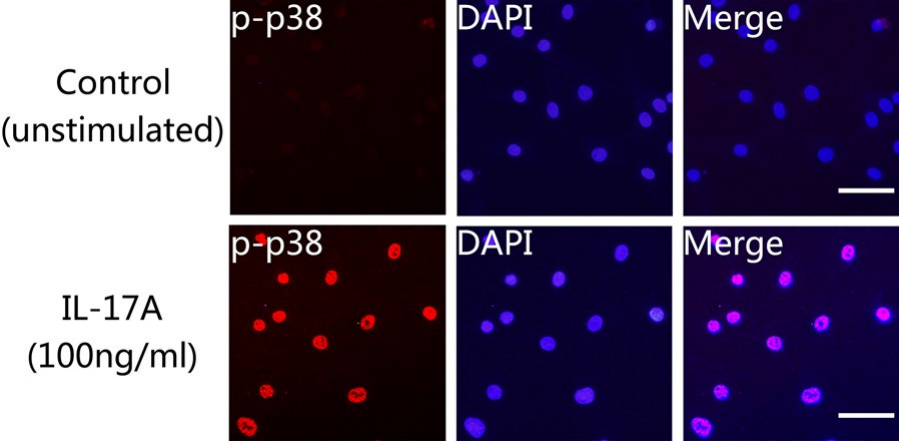
IL-17A induces phosphorylation and activation of p38 in NP cells. NP cells were transferred to a 24-well plate and cultured in serum-free medium for 12 h before treatment with IL-17A (100 ng/ml) for 15 min. The cells were then performed to Immunofluorescence staining. NP cells were incubated with primary antibodies against p-p38 and anti-rabbit IgG secondary antibody (TMRITC). DAPI mounting medium was used for nuclear staining. *Left*: cells stained with antibody to p-p38 (*red*). *Middle*: cells stained with DAPI to identify nuclei, (*blue*). *Right*: cells stained with antibody to p-c-Jun and with DAPI. The results revealed that phosphorylation level of p38 was promoted significantly in IL-17A treated NP cells than in untrated controls. *Scale bar* = 50 μm

To evaluate signalling pathway responses in NP cells activated by IL-17A, the MAPK pathway was examined. The expression levels of p-p38, P38, p-JNK, JNK, p-ERK1/2 and ERK1/2 were analysed using western blots. The results demonstrated that IL-17A induced transient activation of the p38 MAPK pathway, resulting in elevated phosphorylation of p38 MAPK at15 min, which then phosphotylation level decreased, with levels remaining detectable through 120 min (Fig. 2a, d). What’s more, the immunofluorescence test of p-p38 showed that phosphorylation of p38 can be induced by IL-17A immediately (Fig. 3). Increased phosphorylation of JNK occurred within 15 min of IL-17A treatment and reached a peak level by 60 min, remaining at a level higher than basal at 120 min (Fig. 2b, e). In contrast, the ERK/MAPK pathway was hardly activated following exposure to IL-17A, as the phosphorylation of ERK1/2 was not detectable even at 120 min (Fig. 2c, f).

### Regulation of COX2/PGE2 in response to IL-17A

**Fig. 4:**
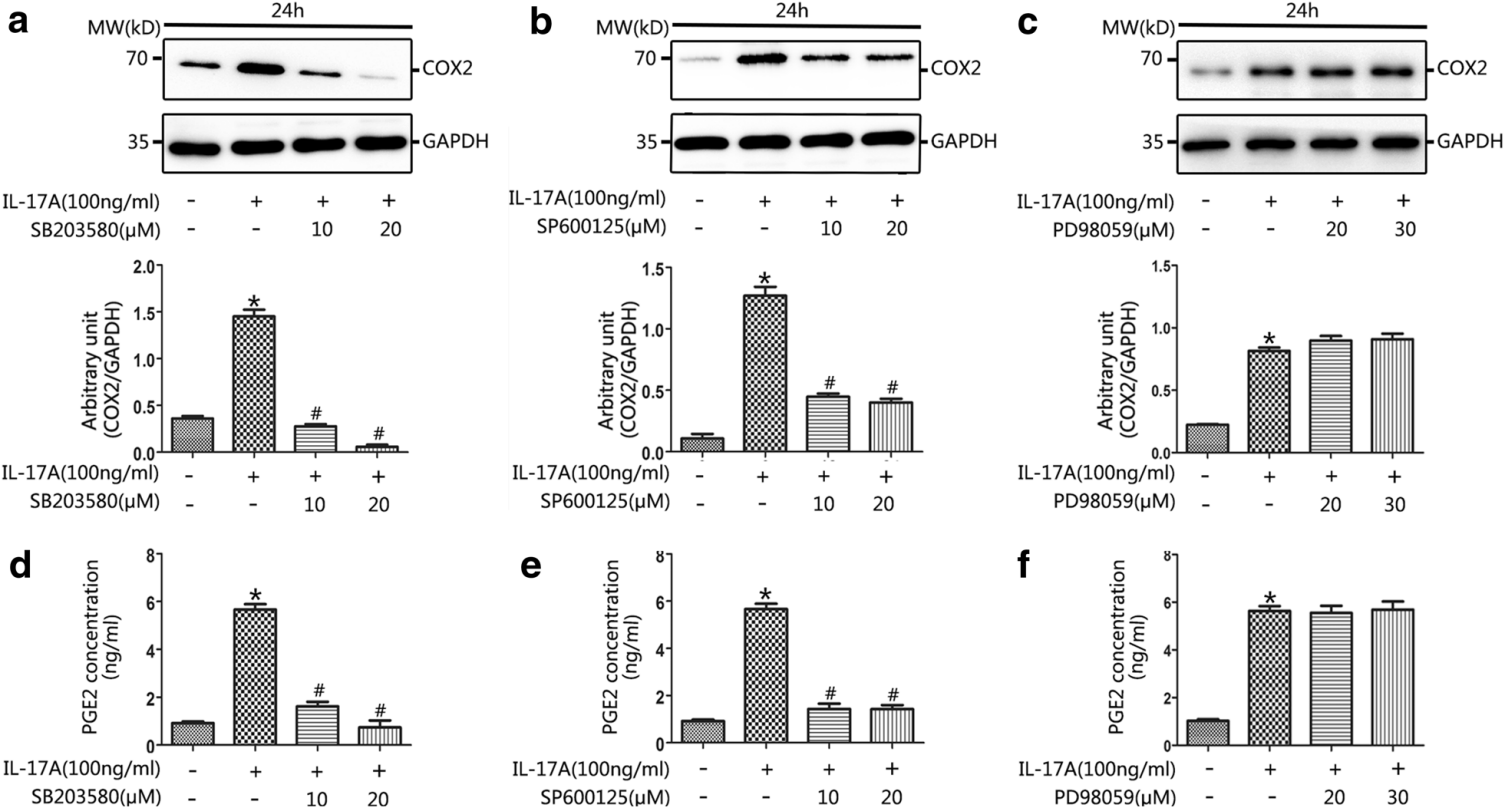
IL-17A enhanced COX2 and PGE2 expression was associated with increased p38 and JNK signaling. NP cells were cultured in serum-free medium prior to treatment with SB203580 (10 or 20 μM; p38 MAPK phosphorylation inhibitor), SP600125 (10 or 20 μM; JNK MAPK phosphorylation inhibitor), or PD98059 (20 or 30 μM; ERK1/2 MAPK phosphorylation inhibitor) for 30 min prior to the addition of IL-17A (100 ng/ml) for 24 h. The cell lysates were prepared for western blot of COX2 and cell-free supernatants were harvested for ELISA of PGE2. Pretreatment with SB203580 (10 or 20 μM), SP600125 (10 or 20 μM) for 30 min prior to IL-17A induction for 24 h in NP cells significantly declined the expression of COX2 (**a**, **b**) and PGE2 (**d**, **e**) compared with IL-17A-only treatment (*P < 0.01, vs. control; ^#^P < 0.001, vs. IL-17-only treatment). However, the levels of COX2 (**c**) and PGE2 (**f**) were with no significant difference in the presence of PD98059 (20 or 30 μM) after IL-17A-induced MAPK stimulation (*P < 0.01, vs. control)

To investigate the involvement of the MAPK pathway in the regulation of COX2 and PGE2 levels following exposure to IL-17A, primary NP cell cultures were treated with pharmacological MAPK inhibitors. The P38 pathway was specifically blocked with SB203580, the JNK pathway with SP600125, and the ERK pathway with PD98059. Immunoblot analysis showed that inhibition of the P38/MAPK pathway (using 10 or 20 μM SB203580) or the JNK/MAPK pathway (using 10 or 20 μM SP600125) significantly suppressed the IL-17A-stimulated upregulation of COX2 (Fig. 4a, b). Combination inhibition of both SB203580 and SP600125 were also performed, the results revealed that inhibition of both P38/MAPK pathway and JNK/MAPK pathways significantly decreased the expression of COX2 (Fig. 6b). In contrast, the upregulation of COX2 by IL-17A appeared to be independent of the ERK/MAPK pathway, as the elevated level of COX2 was unaffected by selective inhibition of the ERK pathway (using 20 or 30 μM PD98059) (Fig. 4c). Similar trends were observed in the PGE2 ELISA results when using selective P38, JNK and ERK MAPK pathway inhibitors (Figs. 4d–f, 6b). These results suggest that the P38/MAPK and JNK/MAPK pathways regulate COX2 and PGE2 expression in primary NP cells.

### IL-17A-mediated upregulation of COX2 and PGE2 in primary NP cells via the P38/c-Fos and JNK/c-Jun signalling pathways

**Fig. 5:**
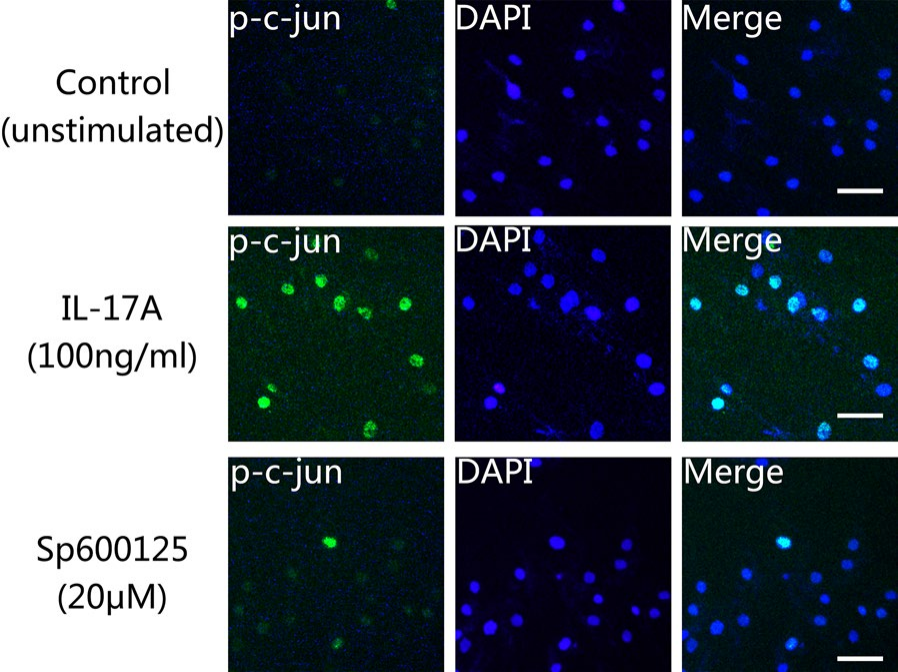
IL-17A induces phosphorylation of c-Jun in NP cells. Primary NP cells were transferred to a 24-well plate and cultured in serum-free medium for 12 h before treatment with SP600125 (20 μM; JNK MAPK phosphorylation inhibitor) for 30 min prior to the addition of IL-17A (100 ng/ml) for 4 h. The cells were performed to Immunofluorescence staining. NP cells were incubated with primary antibodies against p-c-jun and anti-rabbit IgG secondary antibody (FITC). DAPI mounting medium was used for nuclear staining. *Left*: cells stained with antibody to p-c-Jun. *Middle*: cells stained with DAPI to identify nuclei, (*blue*). *Right*: cells stained with antibody to p-c-Jun and with DAPI. The phosphorylation level of c-Jun was promoted strongly in IL-17A treated NP cells than in untrated controls, and the phosphorylation induced by IL-17A can be attenuated by JNK inhibitor SP600125 (20 μM). *Scale bar* = 50 μm

**Fig. 6:**
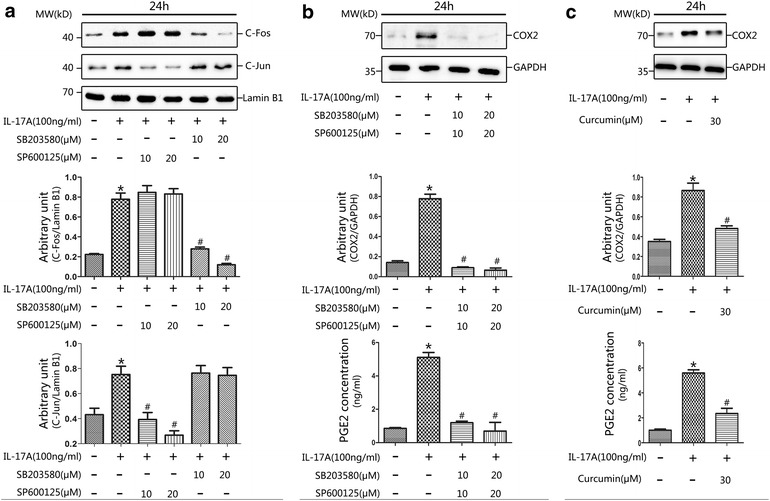
Regulation of COX2 and PGE2 expression is dependent on p38/c-fos and JNK/c-Jun activities. **a** Effects of IL-17A and MAPK inhibitors on IL-17A induced c-Jun and c-Fos translocation. Nucleoprotein was isolated from NP cells treated with SB203580 (10 or 20 μM), or SP600125 (10 or 20 μM) for 30 min prior to IL-17A (100 ng/ml) induction for 24 h. Afterward, western blot analysis was performed using specific antibodies against c-Jun and c-Fos. Expression of lamin B1 was examined as a reference. The *upper panel* suggested that the JNK inhibitor SP600125 effectively inhibited IL-17A-induced c-Jun translocation, while the* lower panel* showed that the p38 inhibitor SB203580 significantly inhibited IL-17A-induced c-Fos translocation. **b** Pretreatment with combination of SB203580 (10 or 20 μM) and SP600125 (10 or 20 μM) for 30 min prior to IL-17A induction for 24 h in NP cells significantly declined the expression of COX2 and PGE2 (*P < 0.01, vs. control; ^#^P < 0.001, vs. IL-17-only treatment). **c** IL-17A-stimulated COX2 and PGE2 expression depends on AP-1 activation. Pretreatment with curcumin (30 μM) for 30 min prior to IL-17A (100 ng/ml) induction for 24 h in NP cells significantly reduced the expression of COX2 and PGE2 compared with IL-17A-only treatment. (*P < 0.01, vs. control; ^#^P < 0.001, vs. IL-17-only treatment)

The results described above indicated that activation of the p38 and JNK MAPK pathways is involved in IL-17A-mediated induction of COX2 and PGE2 expression. Additionally, the COX2 promoter has been shown to contain a motif recognized by the transcription factor AP-1 [42], which is composed of c-Jun and c-Fos [30]. To identify the signalling cascades by which IL-17A stimulated MAPK activation, we first used immunofluorescence staining to detect the phosphorylation of c-Jun. The results revealed that IL-17A appeared to stimulate the phosphorylation of c-Jun and that this process could be weakened by blocking the JNK pathway (Fig. 5). This indicated that IL-17A activates phosphorylation of c-Jun via the JNK/MAPK pathway. Furthermore, we examined the effects of IL-17A on the translocation of AP-1, c-Jun and c-Fos. The results indicated that the translocation of both c-Jun and c-Fos, which are the major downstream components of MAPKs, was significantly upregulated by IL-17A (Fig. 6). To further explore the effect of IL-17A-mediated regulation of AP-1 transactivation, we extracted nuclear protein and examined the abilities of MAPK inhibitors to inhibit AP-1 transactivation using western blotting. We observed that the P38 inhibitor SB203580 (10 or 20 μM) could interfere with nuclear c-Fos translocation in NP cells, while the JNK inhibitor SP600125 (10 or 20 μM) downregulated c-Jun nuclear translocation (Fig. 6a). This suggests that c-Fos and c-Jun translocation was upregulated by P38 and JNK phosphorylation, respectively. To further clarify the involvement of AP-1 in IL-17A-induced COX2/PGE2 expression, NP cells were cultured with IL-17A with or without curcumin (30 µM). Curcumin (30 μM) significantly downregulated COX2/PGE2 expression (Fig. 6c). Based on these data, we deduced that stimulation of COX2 and PGE2 expression by IL-17A requires activation of P38/c-Fos and JNK/c-Jun via the AP-1 signalling pathway.

## Discussion

It has been shown that inflammation plays a vital role in the pathogenic processes underlying painful IVD degenerative diseases [6, 11, 43, 44]. Furthermore, previous studies have reported that Th17 cells are recruited to herniated disc tissues and produce IL-17A, stimulating pro-inflammatory processes [5]. Other studies of IVD cells have demonstrated that IL-17, either alone or in combination with TNF-α and IFNγ, can trigger the production of inflammatory stimulators, including NOx, IL-6 and PGE2 [41]. The results of our study demonstrated that human NP cells respond to IL-17A by increasing COX2 and PGE2 production. One of the pro-inflammatory effects of IL-17A depends on its capacity to enhance the expression of COX2, which plays a pivotal role in upregulation of PGE2. In IVD herniation and other degenerative disc diseases, abundant production of PGE2 is considered to be the main cause of LBP and sciatica [18]. In the current study, a dramatic increase in COX2 expression and PGE2 release was observed in NP cells following treatment with IL-17A, which suggests that IL-17A has a key role in the inflammatory process underlying IVD disease.

To date, the intracellular mechanism of IL-17A-mediated regulation of COX2 expression and subsequent PGE2 release in NP cells has not been elucidated. Previous studies have implied that IL-17 plays an important role in multiple sclerosis via the MAPK pathway [36, 45]. Additional roles have been suggested for IL-17 in human pulpitis [46] and glioma [38]. In the present study, we determined that the MAPK pathway is involved in regulating IL-17A-induced production of COX2 and PGE2 in NP cells. Primary NP cell cultures were treated with 100 ng/ml IL-17A for 24 h. Our results provided evidence that exposure to exogenous IL-17A markedly increased the phosphorylation of p38 and JNK but did not increase the phosphorylation of ERK (Figs. 2, 3). Interestingly, pretreatment with the p38 inhibitor SB203580 and the JNK inhibitor SP600125 effectively attenuated IL-17A-induced COX2 and PGE2 expression (Figs. 4, 6b). This inhibition was not found following pretreatment with the ERK inhibitor PD98059. The role of the MAPK pathway in regulating COX2 and PGE2 production was demonstrated, as attenuation of both p38 and JNK signalling pathways significantly abrogated IL-17A-mediated COX2 and PGE2 expression.

In addition to the NP cells in our study, COX2 expression has been observed to be induced by AP-1, which regulates the expression of several genes, in epithelial cells [42] and fibroblasts [47]. Furthermore, overexpression of c-Fos in NP cells increased the IVD degenerative process [32]. In the present study, we attempted to clarify the possible molecular mechanisms by which IL-17A upregulates the expression of COX2 and PGE2. Interestingly, we observed that IL-17A remarkably promoted the phosphorylation of c-Jun (Fig. 5). As previously reported, c-Jun is not only a downstream transcription factor in the MAPK pathway but also a target of JNK [30]. JNK activation induces the phosphorylation of c-Jun, which then promotes the expression of c-Jun. Moreover, the present study showed that IL-17A induced the nuclear translocation of c-Jun and c-Fos, which could be effectively inhibited by the JNK inhibitor SP600125 or the p38 inhibitor SB203580 (Fig. 6a). Further studies revealed that inhibition of AP-1 activation by curcumin significantly reduced COX2 expression and PGE2 production (Fig. 6c). What’ more, an interesting result was that when either p38/c-Fos or JNK/c-Jun pathway is inhibited, the productions of COX2 or PGE2 were decreased by more than 70 %. We assume the reason was that AP-1 was existed and functioned in the form of heterodimers of c-jun and c-fos, in the expression of COX2, both c-jun and c-fos were needed to form AP-1 heterodimers and activated, then bind to the AP-1 binding site to promote COX2 expression. In summary, we suggest that IL-17A-induced COX2 expression and PGE2 production are AP-1-dependent processes that act through parallel signalling cascades involving p38/c-Fos and JNK/c-Jun.

**Fig. 7:**
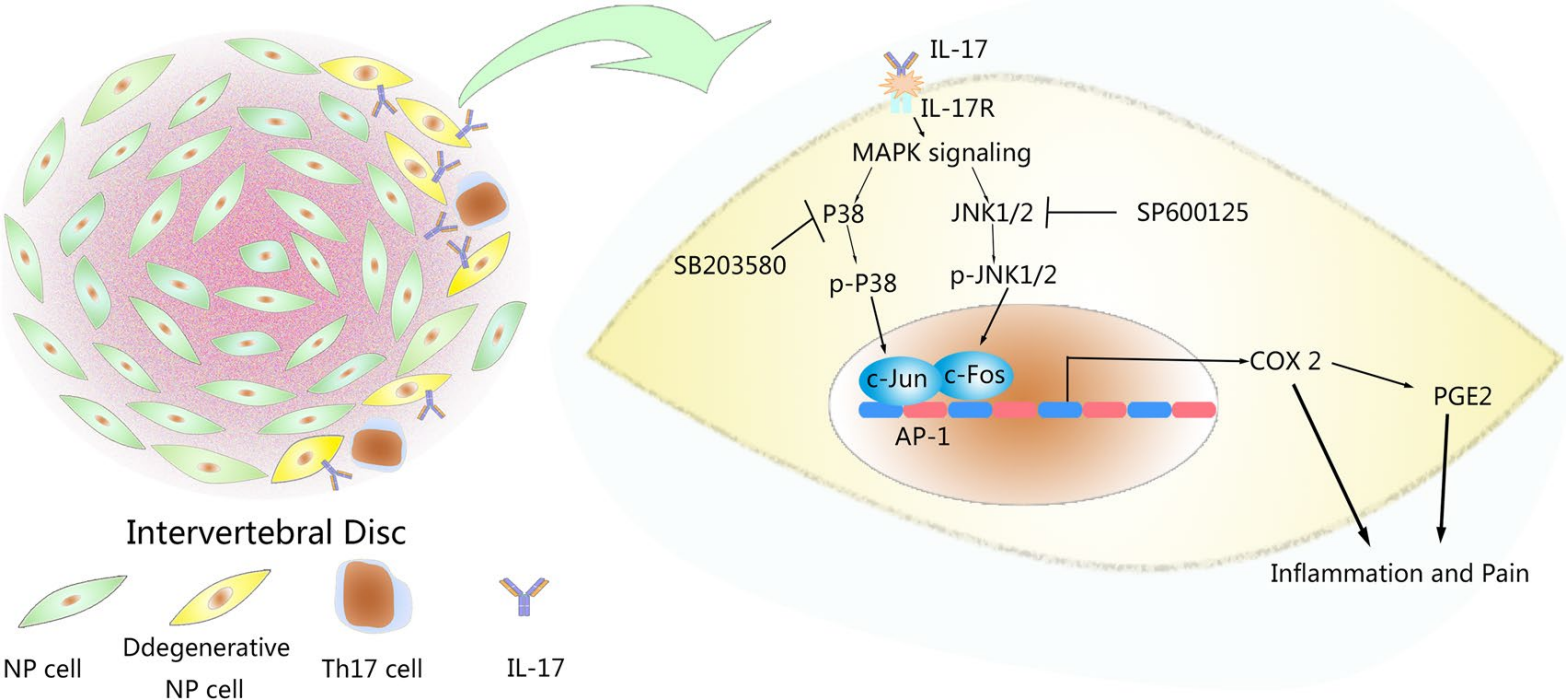
Schematic model of the proposed signal pathways of inflammation induced by IL-17A

Previous studies have shown that Th17 cells contribute to IVD inflammation both directly and indirectly [5, 27, 41]. Based on previous evidence of the pro-inflammatory activities of IL-17A, for the first time, we described a hypothetical model elucidating the potential mechanisms by which IL-17A induces COX2 expression and PGE2 production in NP cells (Fig. 7). In our model, peripheral Th17 cells are transferred to the IVD to secrete IL-17A. In the inflammatory microenvironment, IL-17A activates IL-17 receptors on NP cell membranes and triggers p38 and JNK activation, thereby inducing the phosphorylation of c-Jun and the translocation of c-Fos and c-Jun. C-Fos and c-Jun then cooperatively bind to the AP-1-binding site and promote COX2 expression and subsequent PGE2 production. SB203580 acts as an inhibitor of the p38 signalling pathway, while SP600125 inhibits the JNK pathway, suppressing IL-17A-induced MAPK activation and COX2 expression.

In our study, p38-MAPK and JNK-MAPK inhibition significantly reduced the induction of COX2 expression by IL-17A. This suggests that inhibition of MAPK/AP-1 activation by different pharmacological agents might be useful against IL-17-induced COX2/PGE2 production and IVD inflammation. This may provide a potential therapeutic target to ameliorate IVD inflammation. However, another important factor to be considered is that inhibition of the MAPK pathway might result in severe cytotoxicity in NP cells [6]. Strict screening is necessary before MAPK inhibitors can be used to treat IVD inflammation.

## Conclusion

In conclusion, our results revealed that IL-17A exerts pro-inflammatory effects by increasing COX2/PGE2 production in NP cells through activation of the MAPK pathway in an AP-1-dependent manner.

## Abbreviations

LBP: low back pain
IVD: intervertebral disc
NP: nucleus pulposus
PLIF: posterior lumbar interbody fusion
PBS: phosphate-buffered saline
